# Alterations of the gut microbiota and metabolites by ShenZhu TiaoPi granule alleviates hyperglycemia in GK rats

**DOI:** 10.3389/fmicb.2024.1420103

**Published:** 2024-08-29

**Authors:** Jindong Zhao, Zhaohui Fang

**Affiliations:** ^1^Department of Endocrinology, The First Affiliated Hospital of Anhui University of Chinese Medicine, Hefei, Anhui, China; ^2^Center for Xin'an Medicine and Modernizatison of Traditional Chinese Medicine of IHM, The First Affiliated Hospital of Anhui University of Chinese Medicine, Hefei, Anhui, China

**Keywords:** type 2 diabetes mellitus, alleviating hyperglycemia, gut microbiota, gut metabolites, ShenZhu TiaoPi granule

## Abstract

ShenZhu TiaoPi granule (STG) is a compound prescription that is used in Chinese medicine for the treatment of type 2 diabetes mellitus (T2DM). Previous studies have indicated a hypoglycaemic effect, but the underlying mechanism remains unclear. Goto-Kakizaki (GK) rats were used to establish an in vivo T2DM model (Mod). The metformin (Met) and STG treatment time was 12 weeks. Fasting blood glucose (FBG) and insulin levels and the area under the glucose curve (GAUC) were measured. Intestinal pathology and permeability were observed. Microbial diversity analysis and metabolomics were used to investigate the underlying mechanisms. Compared with the Con group, the T2DM Mod group presented significant differences in weight, FBG, GAUC, and homeostasis model assessment–insulin resistance (HOMA-IR) indices (*p* < 0.01). Met and STG improved these indicators (*p* < 0.01). The pathological morphology and zonula occludens 1 protein levels in the intestines of the Mod group of rats were altered, leading to increases in the lipopolysaccharide (LPS) and interleukin-1β (IL-1β) levels. In the Met and STG groups, the intestinal conditions improved, and the LPS and IL-1β levels significantly decreased (*p* < 0.01). Changes in the gut microbiota and metabolites occurred in the Mod group. In the STG group, the abundance of Intestinimonas increased, and the abundance of Eubacterium coprostanoligenes decreased significantly (*p* < 0.05). Moreover, STG also altered 2-deoxyglucose, beta-muricholic acid and dioxolithocholic acid production. In addition, the main metabolic pathways affected by STG were bile acid biosynthesis and cholesterol metabolism. Intestinimonas, D-maltose_and_alpha-lactose may be potential biomarkers for the effects of STG. STG alleviates hyperglycaemia via the gut microbiota and metabolites in GK rats.

## Introduction

Type 2 diabetes mellitus (T2DM) is a metabolic disease characterized by elevated blood glucose levels due to a complex combination of genetic and environmental factors (Chen and Wang, 2021; Laffel et al., 2023). Good glycemic control has a profound effect on the occurrence, development and regression of T2DM and its acute and chronic complications (Wang et al., 2018). In modern daily life, unhealthy dietary structures and exercise patterns have accelerated the rising trend of T2DM and its rejuvenation, seriously jeopardizing human health (Fujihara et al., 2023).

The intestinal flora is an important internal environment in the body, and a decrease in beneficial bacteria and an increase in pathogenic bacteria in the intestinal tract can lead to the abnormal function of organs, tissues and cells of the body, which can contribute to an imbalance in glucose metabolism (Chen and Wang, 2021). Intestinal metabolites are important signaling factors, energy substrates, nutritional sensors and metabolic regulators involved in the development of T2DM (Swann et al., 2011; Jia et al., 2018).

T2DM belongs to the category of “Xiao Ke” in Chinese medicine. The etiology and pathogenesis of T2DM are qi and yin deficiency. Among them, qi and yin are highly energetic and important substances in the human body. Qi and yin deficiency syndrome can manifest as thirst, increased diet, increased frequency of urination, and weight loss, among other symptoms. Chinese medicine has clinical efficacy in the prevention and treatment of T2DM (Bai et al., 2019). Shenzhu Tiaopi granule (STG) tonifies qi and nourishes yin. Clinical studies have shown that STG can lower blood glucose levels, alleviate dry mouth, thirst, and fatigue, and reduce the frequency of urination (Fang et al., 2014; Zhang et al., 2023). These changes are associated with improved hepatic insulin resistance (Yin et al., 2021). However, whether STG affects the gut flora or metabolites is unclear. The present study was conducted using GK rats with T2DM to further explore the effects of STG in regulating blood glucose levels by affecting the intestinal flora and metabolites and to explore the possible mechanisms of action to provide an effective prescription for alleviating hyperglycaemia in individuals with T2DM and to facilitate scientific research on the prevention and treatment of T2DM by traditional Chinese medicine.

## Materials and methods

### Animals and experimental design

Fifteen-to sixteen-week-old male GK rats were obtained from Changzhou Cavens Model Animal Co., Ltd. (Changzhou, China; certificate no. 202145537). Male Wistar rats of the same age were obtained from Sipeifu (Beijing) Biotechnology Co., Ltd. (Beijing, China; certificate no. 110324210106676238). The rats were acclimated in a specific pathogen-free laboratory. The rats were provided standard chow and water *ad libitum*. The temperature was maintained at 22 ± 2°C with 50–70% humidity on a 12/12 h light/dark cycle.

After acclimation for 1 week, 32 GK rats with high fasting blood glucose (FBG) levels (≥ 11.1 mmol/L) were randomly divided into three groups (*n* = 8). The T2DM model group (Mod) received distilled water intragastrically. Metformin (Met) obtained from Sino-American Shanghai Squibb Pharmaceuticals Ltd. (Shanghai, China) was administered by oral gavage at 100 mg/kg/d to the Met group. The STG group was orally gavaged with 21 g/kg/d STG. Wistar rats in the control (Con) group received distilled water via oral gavage. The course of treatment was 12 weeks.

### Weight and blood glucose measurements

Weight and FBG levels were measured once every 2 weeks. FBG levels were measured as follows: after 12 h of fasting, blood was collected from the tail vein of the rats, and FBG levels were measured using a Roche ACCU-CHEK Performa glucometer (Basel, Switzerland).

After 12 h of fasting, each rat was administered 2.0 g/kg of 50% dextrose by gavage, and blood glucose levels were measured using a blood glucose meter after blood was collected from the tail tip for oral glucose tolerance test (OGTT) measurements before and 15, 30, 60, 90, and 120 min after gavage.

The area under the glucose curve (GAUC) was calculated as 0.5 × (FPG + glucose for 30 min) × 30 + 0.5 × (glucose for 30 min + glucose for 60 min) × 30 + 0.5 × (glucose for 60 min + glucose for 120 min) × 60 (Gu et al., 2019). The GAUC is reported in min-mmol/L.

### Sample collection and biochemical analysis

The rats were weighed and anaesthetized deeply via an intraperitoneal injection of 30 mg/kg pentobarbital sodium (Merck, United States). Blood samples were collected in non-heparinized tubes and centrifuged at 4,000 rpm for 10 min at 4°C. The contents were removed by separating and dissecting the ileum. The serum and ileum contents were stored in Eppendorf tubes at −80°C. The fasting insulin (FIns, American Laboratory Products Company, New Hampshire, United States), interleukin-1β (IL-1β, Abbkine Scientific Co., Ltd., WuHan, China), and lipopolysaccharide (LPS, MyBiosource, Inc., Santiago, United States) concentrations were analyzed using enzyme-linked immunosorbent assay kits according to the manufacturers’ instructions.

The homeostasis model assessment–insulin resistance (HOMA-IR) score was calculated as the FIns level (miu/L) × FBG level (mmol/L)÷22.5 (Hu et al., 2022).

### Histology

The ileum was washed with phosphate-buffered saline, fixed in a tube containing a 4% paraformaldehyde solution for 24 h, embedded in paraffin wax, sliced into 4 μm sections, and stained with hematoxylin and eosin. Intestinal zonula occludens 1 (ZO-1) protein levels were observed through immunofluorescence staining to evaluate the defense barrier function of the intestinal mucosa. Images were captured using a Nikon Eclipse Ci-L microscope (Tokyo, Japan; 200× magnification).

### 16S sequencing of the gut microbiome and metabolomic profiling

Total ileal bacterial DNA was extracted using a MagPure Stool DNA KF kit B (Guangzhou Magen Biotechnology Co., Ltd., Guangzhou, China) according to the manufacturer’s instructions. Approximately 30 ng of DNA was used to generate amplicons with an Agencourt AMPure XP kit (Beckman Coulter, California). After PCR amplification of the full-length 16S rDNA, the qualified library was sequenced on the HiSeq 2,500 platform, which targets the V4 region (California, America) (Zhang et al., 2020). The raw data were filtered to generate high-quality clean reads. Tags were generated using Fast Length Adjustment of Short Reads (v1.2.11). Clustering was performed according to 97% sequence similarity to determine the abundance of operational taxonomic units (OTUs) in the gut microbiota via USEARCH (v7.0.1090) (Zhu et al., 2020).

After the ileal samples were crushed, an HM400 standard curve was generated, and the samples were subjected to derivatization, dilution and other steps prior to analysis. The resulting supernatant was subjected to liquid chromatography–mass spectrometry on a QTRAP 6500 + SCIEX instrument (Massachusetts, America) (Peng et al., 2023). The chromatographic column used was a BEH C18 column (2.1 mm × 10 cm, 1.7 μm, Waters, Massachusetts, America). The ion source was an electrospray ionization (ESI)+/ESI-system. The parameters for the integration of each multiple-reaction monitoring transition and manual inspection were determined with HMQuant software (BGI Shenzhen, Guangdong, China) (Li et al., 2023). The concentration was calculated according to the integrated peak area of the target index (Bai et al., 2023).

### Statistics and analysis

The data were analyzed using one-way analysis of variance followed by the least significant difference test or Dunnett’s T3 test. Partial least squares discriminant analysis (PLS-DA) and principal component analysis (PCA) were used to display classification changes with R software (v3.1.1), mixOmics, and the ade4 package. A Venn diagram and heatmap were constructed with the R software (v3.1.1) Venn diagram and gplots packages. The Kyoto Encyclopedia of Genes and Genomes (KEGG) pathways were predicted using PICRUSt2 v2.2.0-b and R (v3.4.10). ImageGP was used for the intergroup pathway analysis. Significantly enriched metabolite pathways were identified using the MetaboAnalyst platform.^1^ An analysis of functional differences was performed using the Wilcoxon test. The data were analyzed using the Microbial Amplification Subsystem^2^ and the Gene Denovo Cloud Platform.^3^ SPSS 23.0 (New York, United States) and GraphPad Prism 5.0 (San Diego, United States) were used for analyses. An analysis of the receiver operating characteristic (ROC) curves of metabolites is a common method for screening potential biomarkers. The area under the curve (AUC) was calculated using R software (version 4.2.1). When a single factor was used, the pROC package was used to perform the ROC curve analysis, and the results were visualized via ggplot2. Before conducting the joint factor analysis, the data were cleaned, and a multifactor logistic regression model was constructed using a generalized linear model. The model continued the analysis with the same process as before. *p* < 0.05 were considered to indicate statistical significance.

## Results

### Effects of STG on weight and blood glucose-related indicators

**Figure 1:**
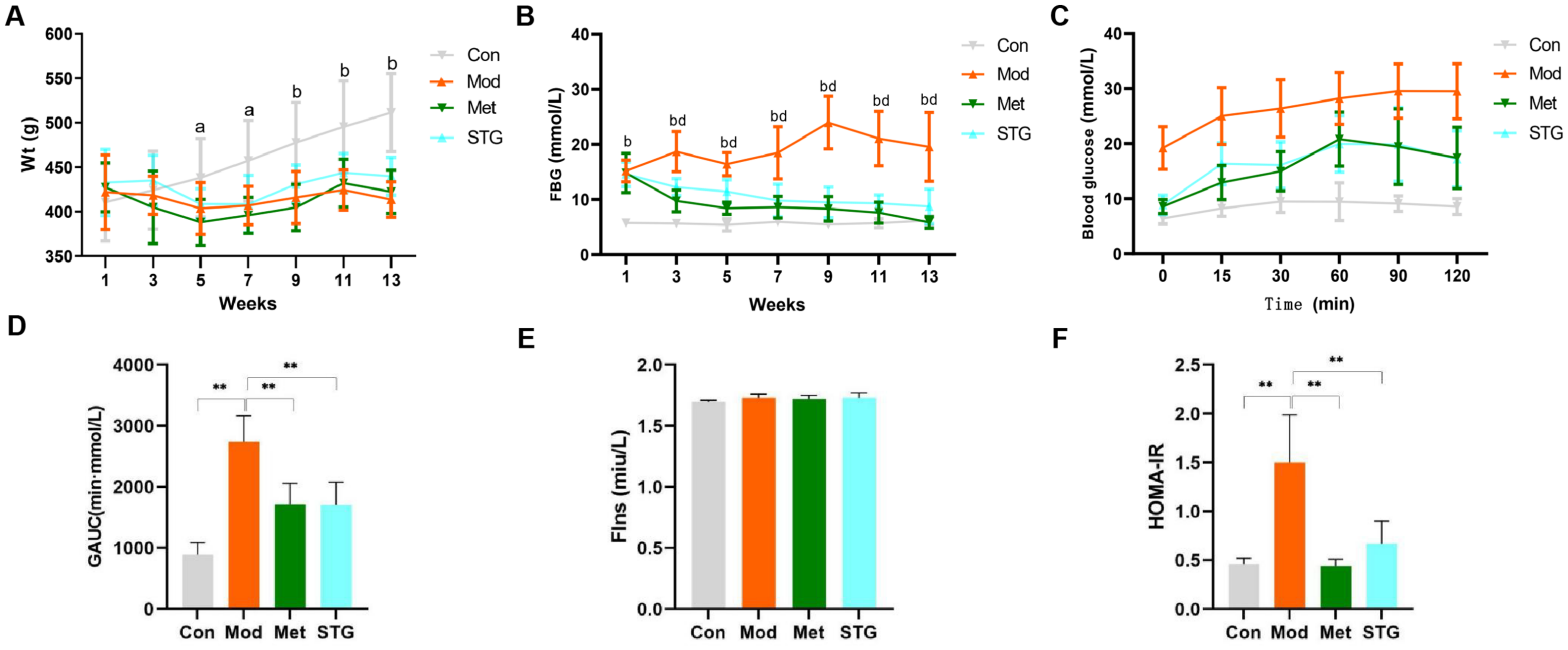
Effects of STG on weight and glucose metabolism-related indicators in rats. **(A)** Comparison of weight. **(B)** Comparison of FBG levels. **(C)** Comparison of blood glucose levels obtained with the OGTT. **(D)** Comparison of the GAUC obtained with the OGTT. **(E)** Comparison of FIns levels. **(F)** Comparison of HOMA-IR. The data are presented as the means ± standard deviations; *n* = 8 rats in the four groups. a = Mod group vs. Con group, *p* < 0.05; b = Mod group vs. Con group, *p* < 0.01; d = Met or STG group vs. Mod group, *p* < 0.01; Con, control; Mod, T2DM model; Met, metformin; STG, Shenzhu Tiaopi granule; T2DM, type 2 diabetes mellitus; Wt, weight; FBG, fasting blood glucose; GAUC, area under the glucose curve; FIns, fasting insulin; HOMA-IR, homeostasis model assessment–insulin resistance. 1, Acclimation for 1 week; 3, the second week after the drug intervention; 5, the fourth week after the drug intervention; 7, the sixth week after the drug intervention; 9, the eighth week after the drug intervention; 11, the tenth week after the drug intervention; and 13: the twelfth week after the drug intervention. **p < 0.01.

Compared with the Con group, the Mod group presented significant reductions in weight at weeks 5, 7, 9, 11, and 13 (*p* < 0.05 or *p* < 0.01). Compared with those in the Mod group, the weight changes in the Met and STG groups were not significantly different (*p* > 0.05). Compared with that in the Met group, the weight change in the STG group was non-significant (*p* > 0.05), as shown in Figure 1A.

Compared with the Con group, the Mod group presented a significant increase in FBG levels at weeks 1, 3, 5, 7, 9, 11, and 13 (*p* < 0.01). Compared with those in the Mod group, the FBG levels in the Met and STG groups decreased significantly (*p* < 0.01). Compared with that in the Met group, the FBG level in the STG group did not change significantly (*p* > 0.05) (Figure 1B). Compared with that in the Con group, the GAUC in the Mod group was significantly greater (*p* < 0.01). Compared with that of the Mod group, the GAUC of the Met and STG groups decreased significantly (*p* < 0.01). Compared with that in the Met group, the GAUC of the STG group did not change significantly (*p* > 0.05) (Figures 1C,D).

No significant difference in the FIns level was observed between the Mod group and the Con group (*p* > 0.05). Compared with that in the Mod group, no significant changes in the FIns levels were observed in the Met and STG groups (*p* > 0.05). Compared with the Con group, the Mod group presented a significant increase in HOMA-IR (*p* < 0.01). Compared with the Mod group, the HOMA-IR index was significantly lower in the Met and STG groups (*p* < 0.01). Compared with those in the Met group, FIns levels and the HOMA-IR in the STG group did not change significantly (*p* > 0.05) (Figures 1E,F).

### Effects of STG on inflammatory indicators and the pathological morphology of the intestine

**Figure 2:**
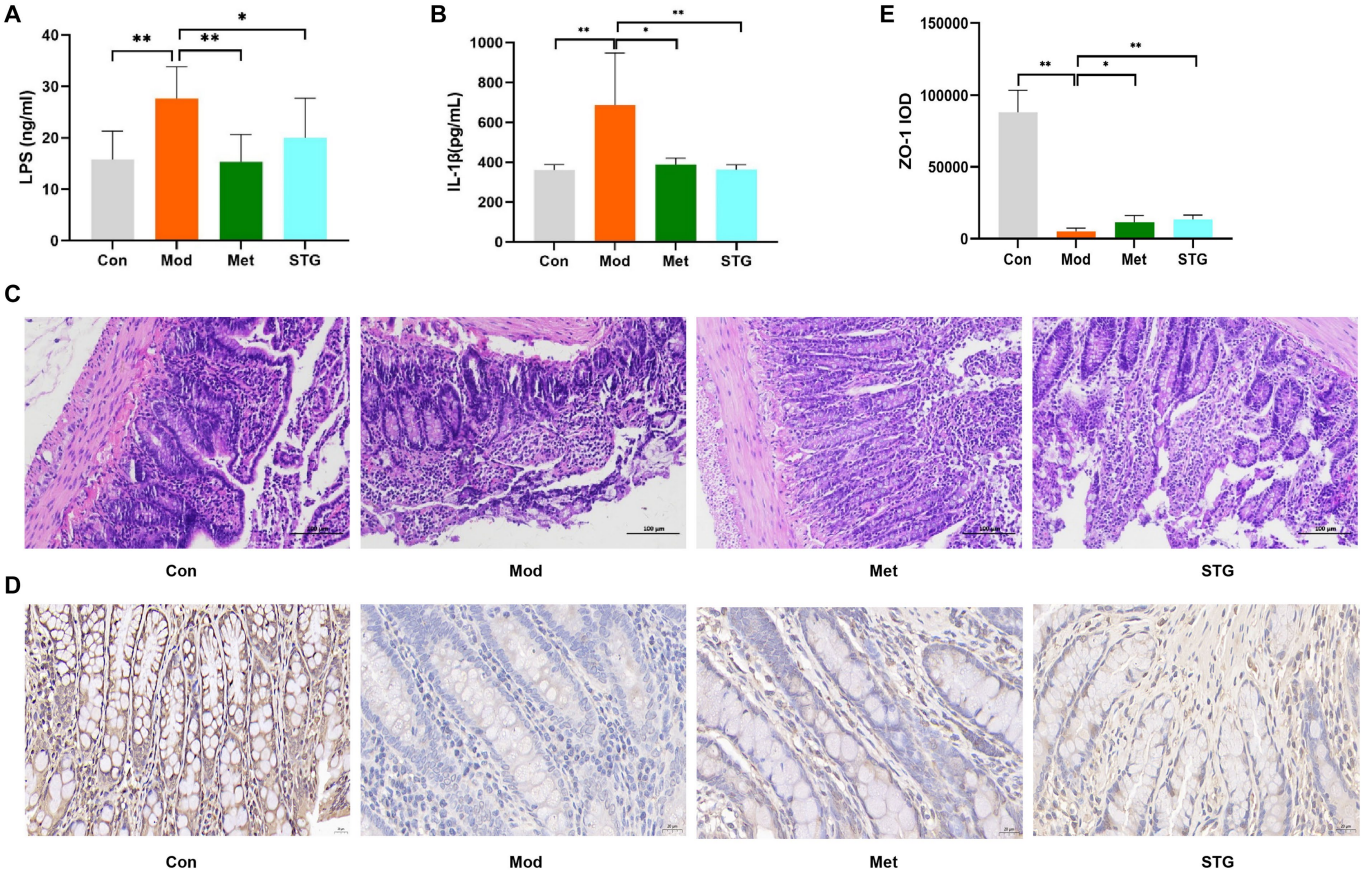
Effects of STG on inflammatory indicators and intestinal pathological morphology in rats. **(A)** Comparison of LPS levels. **(B)** Comparison of IL-1β levels. **(C)** Comparison of HE staining of the ileum. **(D)** Comparison of Zo-1 immunohistochemical staining in the ileum. **(E)** Comparison of the ileal Zo-1 IOD. The data are presented as the means ± standard deviations; *n* = 8 rats in the four groups. Con, control; Mod, T2DM model; Met, metformin; STG, Shenzhu Tiaopi granule; T2DM, type 2 diabetes mellitus; LPS, lipopolysaccharide; IL-1β, interleukin-1β; IOD, integrated optical density. Pathology images are shown separately 200 and 400 × magnification in (**C** and **D**); scale bar, 100 μm and 20 μm in (**C** and **D**). *p < 0.05 and **p < 0.01.

Compared with those in the Con group, the LPS and IL-1β levels in the Mod group were significantly higher (*p* < 0.01). Compared with those in the Mod group, the LPS and IL-1β levels were significantly lower in the Met and STG groups (*p* < 0.05 or *p* < 0.01) (Figures 2A,B).

Compared with the Con group, a decrease in the length of the intestinal villi, an increase in the degree of necrotic shedding of epithelial cells, solidification of the cell nuclei, a smaller volume of intestinal crypts, unclear demarcation, and a small number of cup-shaped cells were observed in the ileum of the Mod group. Compared with the Mod group, the length of the intestinal villi, extent of necrotic shedding of epithelial cells, volume of intestinal crypts, and number of cup-shaped cells were greater in the Met and STG groups (Figure 2C). In addition, the expression of Zo-1 tended to decrease in the Mod group compared with the Con group, whereas Zo-1 protein expression increased in the Met and STG groups (Figure 2D).

### Effects of STG on the gut microbiota community

**Figure 3:**
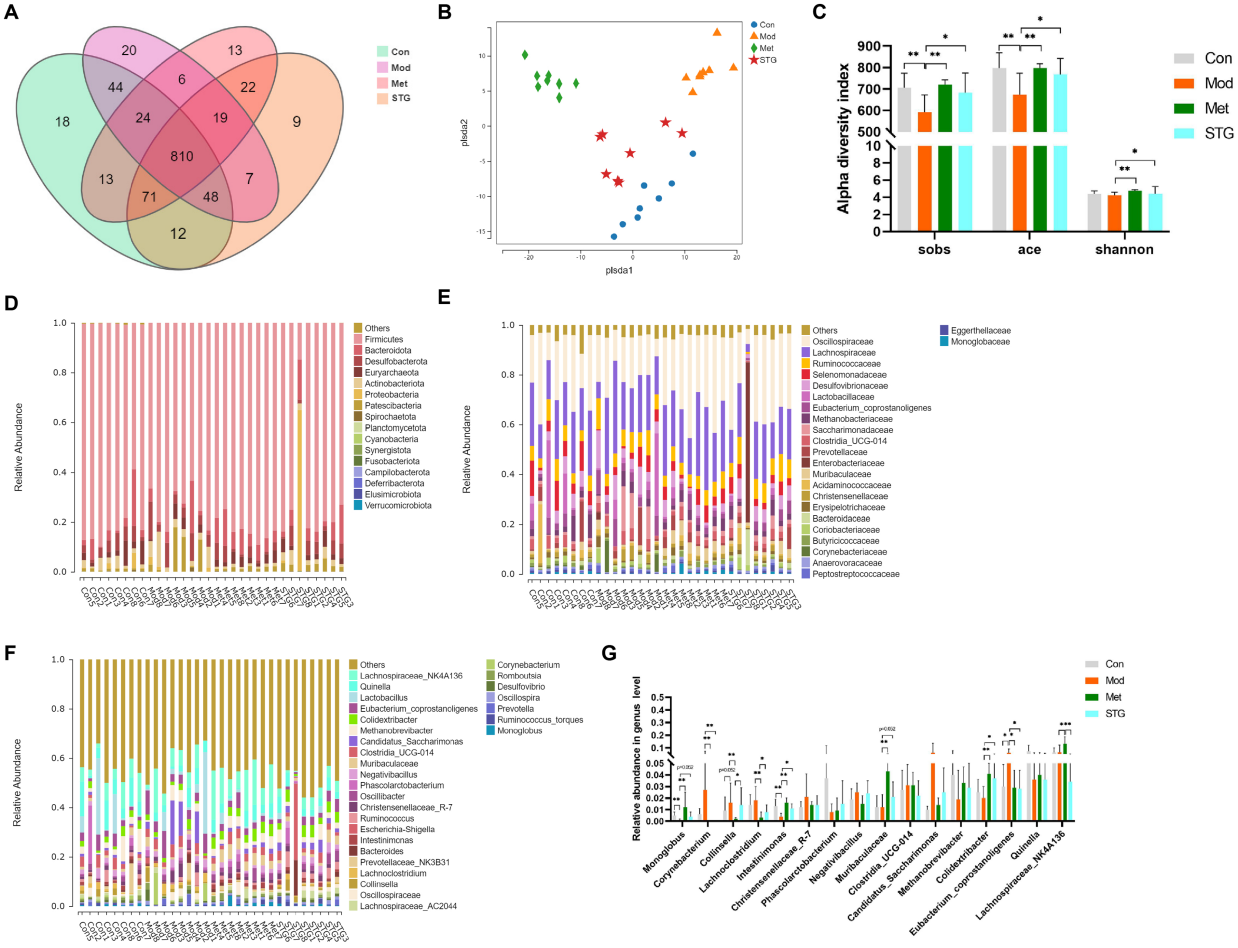
Comparison of the gut microbiota structure. **(A)** Comparison of the OTUs. **(B)** Comparison of the gut microbiota structure using PLS-DA. **(C)** Comparison of alpha diversity. **(D)** Composition at the phylum level. **(E)** Composition at the family level. **(F)** Composition at the genus level. **(G)** Comparison of the relative abundance at the genus level. The data are presented as the means ± standard deviations; *n* = 8 rats in the four groups. OTUs, operational taxonomic units; PLS-DA, partial least squares discriminant analysis. Con, control; Mod, T2DM model; Met, metformin; STG, Shenzhu Tiaopi granule; T2DM, type 2 diabetes mellitus. *p < 0.05 and **p < 0.01.

A total of 148,472 clean reads were obtained, providing a total of 1,136 OTUs. Notably, 9 unique OTUs were identified in the STG group, 13 unique OTUs in the Met group, 20 unique OTUs in the Mod group and 18 unique OTUs in the Con group (Figure 3A). PLS-DA indicated that the clustering of the gut microbiota differed among the four groups. The gut microbiota structure of the mice in the STG treatment group clearly shifted to that of the Con group (Figure 3B). The community richness estimated by the Sobs and ACE indices was significantly lower in the Mod group than in the Con group (*p* < 0.01). Compared with those in the Mod group, the Sobs and ACE indices were significantly higher in the Met and STG groups (*p* < 0.05 or *p* < 0.01). Although no differences in the Shannon index were observed between the Mod and Con groups, the Shannon index was significantly higher in the Met and STG groups than in the Mod group (*p* < 0.05 or *p* < 0.01) (Figure 3C).

The intestinal bacterial composition of each sample at the phylum and family levels is listed in Figures 3D,E. The main components were not significantly different among the four groups (*p* > 0.05). The composition of each sample at the genus level is listed in Figure 3F.

Compared with those in the Con group, the *Monoglobus* and *Intestinimonas* abundances in the Mod group were significantly lower (*p* < 0.01). Compared with those in the Mod group, the abundances of these bacteria were significantly greater in the Met group (*p* < 0.01). In the STG groups, the abundance of *Intestinimonas* increased (*p* < 0.05). Compared with the Con group, the Mod group presented a significant increase in the abundance of *Eubacterium coprostanoligenes* (*p* < 0.05). Compared with that in the Mod group, the abundance of *Eubacterium coprostanoligenes* was significantly lower in the Met and STG groups (*p* < 0.05) (Figure 3G).

### Correlation analysis of the gut microflora with baseline data

**Figure 4:**
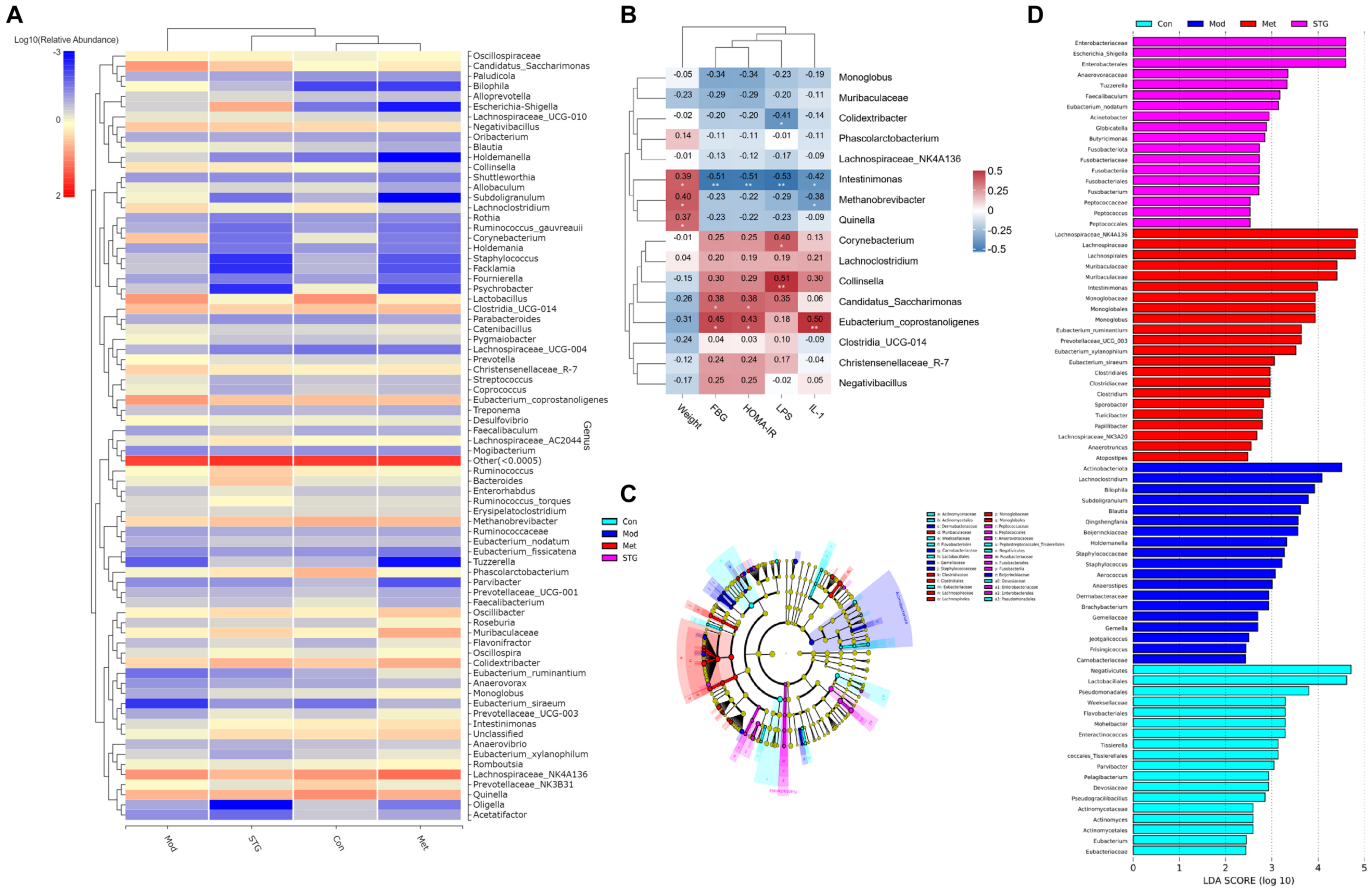
Relationships between the gut microbiota at the genus level and baseline data. **(A)** Clustering heatmap of the relative abundance at the genus level. **(B)** Correlation heatmap of the gut microbiota at the genus level with the baseline data. **(C)** The biomarker of gut microbiota via LDA effect size cluster tree. **(D)** The biomarker of gut microbiota via LDA figure. *N* = 8 rats in the four groups. Con, control; Mod, T2DM model; Met, metformin; STG, Shenzhu Tiaopi granule; T2DM, type 2 diabetes mellitus; FBG, fasting blood glucose; HOMA-IR, homeostasis model assessment–insulin resistance; IL-1β, interleukin-1β; LPS, lipopolysaccharide; LDA, linear discriminant analysis. *p < 0.05 and **p < 0.01.

At the genus level, the clustering heatmap revealed that the Met group was closest to the Con group, followed by the STG group and finally the Mod group (Figure 4A). FBG, HOMA-IR, and IL-1β levels were significantly negatively correlated with *Intestinimonas* abundance (*p* < 0.01). Moreover, the LPS level was significantly positively correlated with *Collinsella* abundance (*p* < 0.01), as shown in Figure 4B. A cladogram showing the relationships among taxa at the phylum, class, order, family, and genus levels was generated using Linear discriminant analysis (LDA) effect size analysis. *Dermabacteraceae*, *Carnobacteriaceae*, *Gemellaceae*, *Staphylococcaceae* and *Beijerinckiaceae* had significant effects on the Mod group. *Muribaculaceae*, *Clostridiaceae*, *Clostridiales*, *Lachnospiraceae*, *Lachnospirales*, *Monoglobaceae* and *Monoglobales* had significant effects on the Met group. *Peptococcaceae*, *Peptococcales*, *Anaerovoracaceae*, *Fusobacteriaceae*, *Fusobacteriales*, *Fusobacteria*, *Enterobacteriaceae* and *Enterobacterales* had significant effects on the STG group (Figure 4C). The components of the gut microbiota with an LDA score > 4 were defined as unique. At the genus level, *Actinobacteriota* and *Lachnoclostridium* were significantly enriched in the Mod group (*p* < 0.01). *Lachnospiraceae_NK4A136*, *Lachnospiraceae*, *Lachnospirales, Muribaculaceae*, and *Muribaculaceae* were significantly enriched in the Met group (*p* < 0.05 or *p* < 0.01). *Enterobacteriaceae*, *Escherichia_Shigella* and *Enterobacterales* were significantly enriched in the STG group (*p* < 0.01), as shown in Figure 4D.

### The functional annotation of the gut microflora

**Figure 5:**
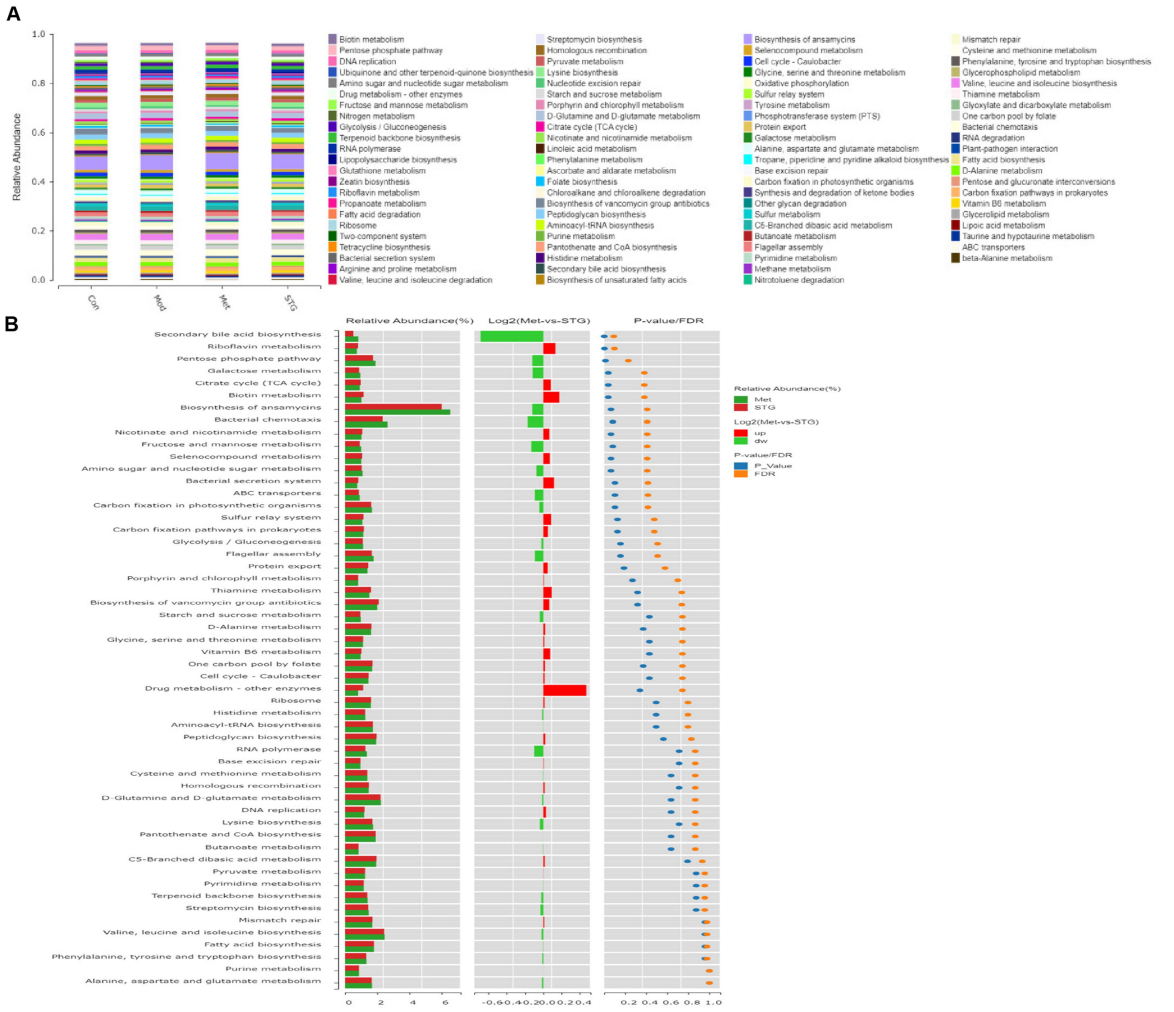
Functional annotation of the gut microbiota. **(A)** KEGG pathway functional annotations at the third level. **(B)** Analysis of differences in KEGG pathway functional annotations at the third level. *N* = 8 rats in the four groups. Con, control; Mod, T2DM model; Met, metformin; STG, Shenzhu Tiaopi granule; T2DM, type 2 diabetes mellitus.

KEGG pathway enrichment analysis was performed to obtain 90 pathways at the third level with an abundance ≥0.0025. Moreover, carbohydrate metabolism-related pathways included amino sugar and nucleotide sugar metabolism, starch and sucrose metabolism, glycolysis/gluconeogenesis, pyruvate metabolism, galactose metabolism, the pentose phosphate pathway, fructose and mannose metabolism, glyoxylate and dicarboxylate metabolism, biotin metabolism, butanoate metabolism, pentose and glucuronate interconversions, the citrate cycle (TCA cycle), propanoate metabolism, and ascorbate and aldarate metabolism (Figure 5A). An analysis of different functions was conducted using the Met and STG groups, and pathways with significant differences in abundance ≥0.8 were identified, including secondary bile acid (BA) biosynthesis, riboflavin metabolism, the pentose phosphate pathway, galactose metabolism, the TCA cycle, and biotin metabolism (Figure 5B).

### Analysis of metabolites in the ileal contents and their functions

**Figure 6:**
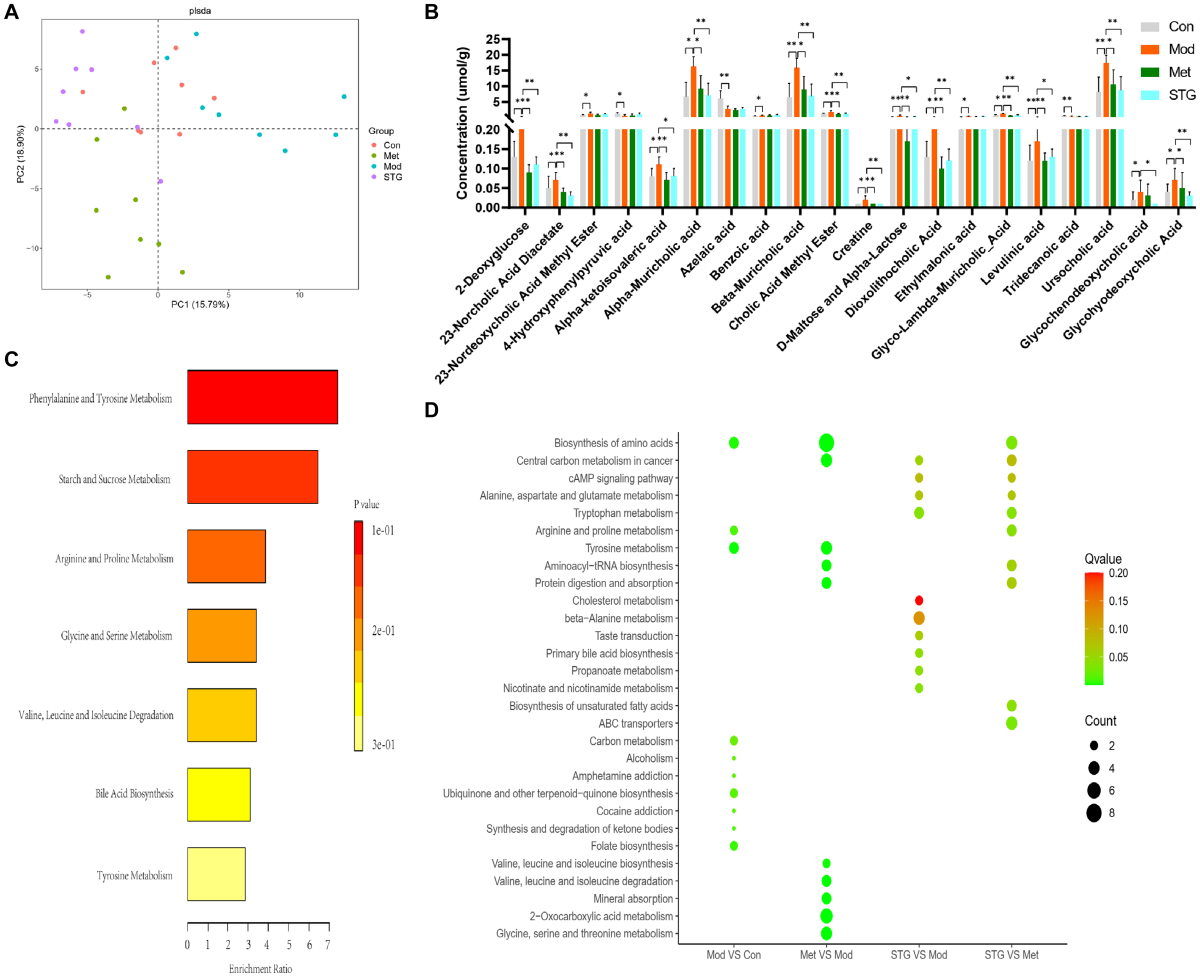
Differentially abundant metabolites in the ileal contents and their functions. **(A)** Comparison of the overall classification effect of metabolites in the ileum contents. **(B)** Comparison of differentially abundant metabolites in the ileal contents. **(C)** Metabolic enrichment analysis of differentially abundant metabolites in the ileal contents. **(D)** KEGG metabolic pathway enrichment analysis of differentially abundant metabolites in the ileal contents. *N* = 8 rats in the four groups. Con, control; Mod, T2DM model; Met, metformin; STG, Shenzhu Tiaopi granule; T2DM, type 2 diabetes mellitus. *p < 0.05 and **p < 0.01.

A total of 403 metabolites were identified in HW400. In this study, 289 metabolites were detected, including 4 pyridines, 7 phenylpropanoids, 3 mixed peptides, 2 organic oxides, 33 organic acids, 7 indoles, 1 imidazole, 55 fatty acids, 9 carnitines, 12 carbohydrates, 33 benzene ring-type compounds, 63 amino acids, 59 BAs, and 1 nucleotide. A comparison of the above metabolites revealed that the overall categorization of the Mod group and the Con group was significant. The Met and STG groups were significantly different from the Mod group. The overall categorization of the Met group with the STG group significantly differed from that of the Mod group, as shown in Figure 6A.

The first 20 metabolites with significant differences were screened and included 3 organic acids, 2 fatty acids, 2 carbohydrates, 2 benzene ring-type compounds, 1 amino acid, and 10 BAs (*p* < 0.05 or *p* < 0.01), as shown in Figure 6B. The top 20 differentially abundant metabolites were enriched mainly in phenylalanine and tyrosine metabolism, starch and sucrose metabolism, and BA biosynthesis, as shown in Figure 6C. The KEGG metabolic pathway enrichment analysis revealed that, compared with the Mod group, the STG group was enriched mainly in primary BA biosynthesis, cholesterol metabolism, nicotinate and nicotinamide metabolism, the cAMP signaling pathway, and tryptophan metabolism, among others, as shown in Figure 6D.

### Correlation analysis of ileal metabolite levels with baseline data and the gut microbiota

**Figure 7:**
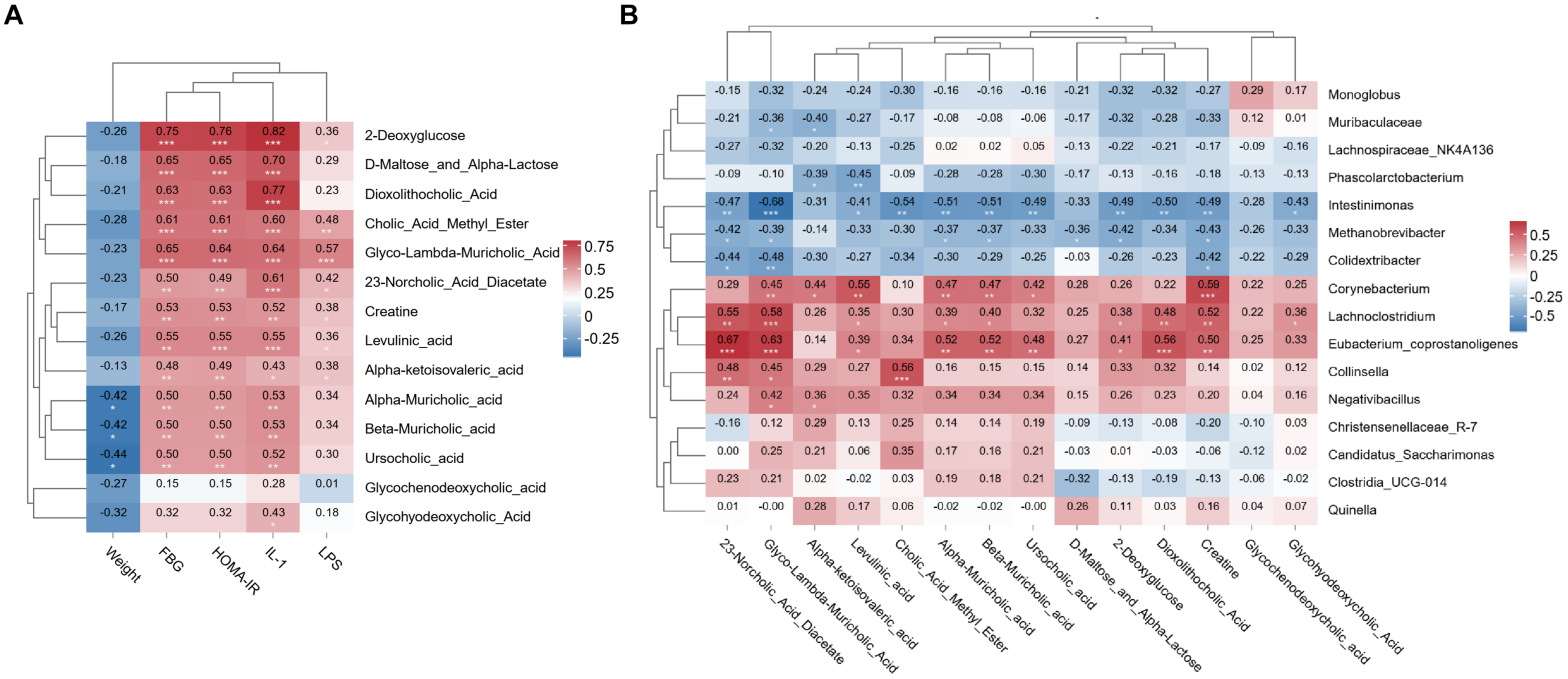
Relationships between ileal metabolite and baseline data or gut microbiota. **(A)** Correlation heatmap of the ileal metabolite contents with the baseline data. **(B)** Correlation heatmap of the ileal metabolite contents with the gut microbiota. *N* = 8 rats in the four groups. FBG, fasting blood glucose; HOMA-IR, homeostasis model assessment-insulin resistance; IL-1β, interleukin-1β; LPS, lipopolysaccharide. *p < 0.05; **p < 0.01; and ***p < 0.001.

The correlation heatmap revealed that FBG, HOMA-IR and IL-1β levels were significantly positively correlated with 2-deoxyglucose, D-Maltose_and_Alpha-Lactose, dioxolithocholic acid, cholic acid methyl ester, creatine, levulinic acid, alpha-muricholic acid, beta-muricholic acid and glyco-lambda-muricholic acid levels (*p* < 0.01). The LPS level was significantly positively correlated with the glyco-lambda-muricholic acid level (*p* < 0.01), as shown in Figure 7A.

The correlation heatmap showed that creatine and levulinic acid levels were significantly positively correlated with *Corynebacterium* abundance (p < 0.01). 23-Norcholic acid diacetate, glyco-lambda-muricholic acid, and creatine levels were significantly positively correlated with *Lachnoclostridium* abundance (*p* < 0.01). 23-Norcholic acid diacetate, glyco-lambda-muricholic acid, alpha-muricholic acid, beta-muricholic acid, dioxolithocholic acid and creatine levels were significantly positively correlated with *Lachnoclostridium* abundance (*p* < 0.01). Glyco-lambda-muricholic acid, cholic acid methyl ester, alpha-muricholic acid, beta-muricholic acid and dioxolithocholic acid levels were significantly negatively correlated with *Intestinimonas* abundance (*p* < 0.01). Moreover, the correlation coefficient between glyco-lambda-muricholic acid and *Intestinimonas* was-0.68, which was the most relevant correlation, as shown in Figure 7B.

### Potential biomarkers in the ileal contents

**Figure 8:**
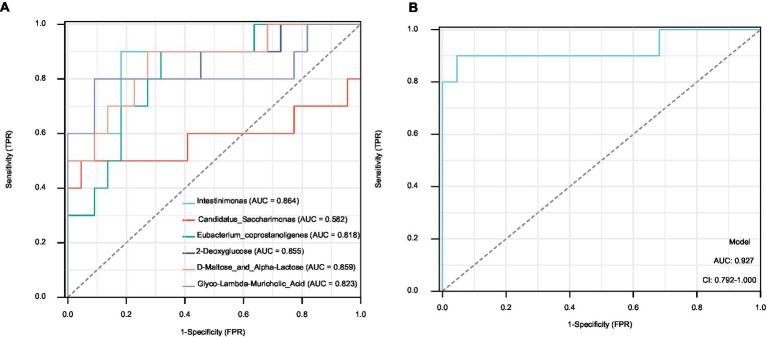
Potential biomarkers in the ileal contents. **(A)** The AUCs of the top three components of the gut microbiota and metabolites. **(B)** The AUCs of D-Maltose_and_Alpha-Lactose and *Intestinimonas*. AUC, area under the curve.

We screened the top three components of the gut microbiota and metabolites that were most strongly correlated with FBG and FIns levels: *Intestinimonas*, *Candidatus_Saccharimonas*, *Eubacterium coprostanoligenes*, 2-deoxyglucose, D-Maltose_and_Alpha-Lactose and glyco-lambda-muricholic acid. The maximum AUC of *Intestinimonas* in the gut microbiota was 0.864. The maximum AUC of D-Maltose_and_Alpha-Lactose was 0.859 for the gut metabolites (Figure 8A). Based on the joint diagnostic analysis of the two highest AUCs, we found that the AUCs of D-Maltose_and_Alpha-Lactose and *Intestinimonas* further increased, with a value of 0.927. These factors may be potential biomarkers for the effect of STG on ameliorating T2DM (Figure 8B).

## Discussion

In individuals with T2DM, the body is in a long-term hyperglycemic state, which aggravates impaired glucose uptake and utilization, exacerbates lipolysis, and is prone to negative protein metabolism equilibrium, as observed in rats in the Mod group, resulting in a significant reduction in weight. This result may be related to the fact that the aging stage of Wistar rats is 24 to 30 months, whereas 30-month-old Wistar rats are in a stage of weight gain. No statistically significant difference in the improvement in weight reduction was observed between the Met and STG groups in the present study. Studies by Wachal et al. (2020) revealed that Met also did not significantly ameliorate the reduction in weight in GK rats. This finding may be related to the fact that Met can reduce fat synthesis in the body and cause adverse reactions in the digestive tract (Haddad et al., 2023; Naseri et al., 2023). Huanglian, the main component of STG, can inhibit fatty acid biosynthesis and adipocyte differentiation and reduce visceral fat or upregulate the expression and secretion of growth differentiation factor-15 mRNA in brown fat, which can reduce weight (Urasaki and Le, 2022; Li et al., 2023).

The blood glucose levels of GK T2DM rats in the Mod group were higher than those in the Con group, whereas the blood glucose levels were lower in the STG group. This finding is consistent with our team’s previous finding that STG can reduce blood glucose levels (Yang et al., 2023). In GK T2DM rats, a significant difference in FIns levels was not observed among the Con, Mod, Met, and STG groups. Moreover, the HOMA-IR in the Mod group was significantly higher than that in the Con group, indicating the occurrence of insulin resistance in GK rats. This result is consistent with the findings reported by Li et al. (2023) and Skoug et al. (2024). However, in the Met and STG groups, the improvement in the HOMA-IR was significantly reduced.

In the Mod group, the ileum showed a reduction in the length of the intestinal villi, a decrease in the volume of the intestinal crypts, and an increase in the shedding of intestinal epithelial cells, resulting in varying degrees of damage to the integrity of the intestinal epithelium and the intestinal barrier. This phenomenon is basically consistent with the findings of Pereira et al. (2021) and Esteves-Monteiro et al. (2022). Both Met and STG improved the intestinal epithelium and the intestinal barrier. A leaky intestine has attracted attention in individuals with T2DM (Sato et al., 2017). In T2DM patients, intestinal permeability is reduced (Sato et al., 2017). The increased blood levels of LPS may be related to an increase in intestinal permeability. LPS activates Toll-like receptor 4, resulting in chronic low-grade inflammation mediated by cytokines such as interferon-γ, tumor necrosis factor-α and IL-1β (Chen et al., 2021). Furthermore, this inflammation promotes the occurrence of T2DM (Beutler, 2004). ZO-1 is an intestinal tight junction protein that reflects intestinal permeability. Decreased ZO-1 levels indicate some damage to the intestinal barrier (González-Mariscal et al., 2011). Our research revealed the malfunction caused by increasing the amount of LPS in the blood. Moreover, the body may develop an inflammatory response. Elevated levels of IL-1β were also observed in this study. *In vivo*, a significant increase in ZO-1 expression was observed in the Met group (Rindone et al., 2024). Moreover, we found that Met could decrease the levels of IL-1β and LPS (Balakumar et al., 2018; Deng et al., 2018). Moreover, STG can improve the levels of ZO-1, IL-1β, and LPS to alleviate T2DM.

The abundance of *Intestinimonas* decreased in the Mod group; however, improvements were observed after the Met and STG interventions. Our research revealed that *Intestinimonas* is a potential biomarker for diagnosing diabetes. The results of another study also showed that verbascoside induced an enrichment of *Intestinimonas* to alleviate glucose metabolism disorders (Ran et al., 2023). The development of Alzheimer’s disease and T2DM are associated with insulin resistance. The *Monoglobus* abundance decreased in the Mod group; however, improvements were observed after Met and STG interventions. *Monoglobus* are short-chain fatty acid-producing bacteria (Verhaar et al., 2022), and the maintenance of the gut and T2DM are related to short-chain fatty acids (Blaak et al., 2020). *Eubacterium coprostanoligenes* is closely related to T2DM (Tsai et al., 2022; Yin et al., 2022). In this study, this relationship was positively correlated with FBG levels. STG decreased the relative abundance of *Eubacterium coprostanoligenes*. In our study, *Candidatus_Saccharimonas* was positively correlated with FBG. *Candidatus_Saccharimonas* is an opportunistic pathogen. In individuals with diabetes, its abundance is elevated (Ye et al., 2024). This result is consistent with our research findings. The abundance of *Enterobacteriaceae* is higher in T2DM patients (Feng et al., 2022). *Enterobacterales* is negatively associated with T2DM (Hernández-Montoliu et al., 2023). However, STG can effectively regulate the abundances of *Enterobacteriaceae* and *Enterobacterales* to alleviate T2DM.

The abundance of *Muribaculaceae* is abnormal in the T2DM model, and *Muribaculaceae* can migrate to the pancreas, where it causes inflammation and beta cell damage (Yang et al., 2023; Song et al., 2024). The abundance of *Lachnospirales* had a significant negative correlation with T2DM. In this study, Met improved the abundances of *Muribaculaceae* and *Lachnospirales*. This result is different from the impact of STG on the gut microbiota. Based on the predicted function of the gut microbiota, STG may improve glucose levels by affecting metabolic pathways. The pentose phosphate pathway plays an important role in T2DM by regulating the effects of glucose 6-phosphate on glycolysis and gluconeogenesis (Ge et al., 2020). Increased levels of TCA cycle metabolites are a risk factor for T2DM (Guasch-Ferré et al., 2020).

Based on the metabolites detected in the ileal contents, differences were observed in organic acids, benzene ring-type compounds, and BAs. Organic acids have a blood glucose-lowering effect (Jamrozik et al., 2022). Alpha-ketoisovaleric acid is related to T2DM (Fujiwara et al., 2021). We found that the alpha-ketoisovaleric acid content was increased in the Mod group. Both Met and STG improved this change. The ethylmalonic acid content was increased in the Mod group, but another study demonstrated that the ethylmalonic acid level was negatively associated with the HOMA-IR (Liu et al., 2020). The levulinic acid content was higher in the Mod group than in the Con group, and a positive correlation was observed between the levulinic acid content and both FBG levels and the HOMA-IR. Both the Met and STG groups exhibited an improved levulinic acid concentration. In addition, the levulinic acid content was positively correlated with *Corynebacterium*. An increase in *Corynebacterium* abundance can lead to an increased risk of diabetic eye disease and diabetic foot infections (Hartemann-Heurtier and Senneville, 2008; Aoki et al., 2021). 2-Deoxyglucose is a carbohydrate, and its content was positively correlated with FBG levels and the HOMA-IR. The AUC of the potential biomarkers for T2DM was 0.855. This result may be related to decreased 2-deoxyglucose uptake into the intestine (Kobayashi, 2018). This compound inhibits the glycolytic pathway and accelerates gluconeogenesis, leading to an increase in blood glucose levels (Thomas and Lowy, 1992). In a diabetic mouse model, 2-deoxyglucose was shown to affect glycine and serine metabolism (Zhang et al., 2022). This phenomenon was also observed in our study. The glycine level is associated with a decreased risk of incident T2DM (Adeva-Andany et al., 2018). Low systemic serine levels are also emerging as a hallmark of diabetes-related peripheral nerve disorders (Handzlik et al., 2023). In addition, D-maltose and alpha-lactose, which are carbon sources, also have effects on blood glucose levels, similar to 2-deoxyglucose (Wang et al., 2023). Moreover, they can be used for the auxiliary diagnosis of T2DM, and their AUC is 0.859. D-Maltose_and_Alpha-Lactose and *Intestinimonas* can be considered potential candidate biomarkers for T2DM. Glyco-lambda-muricholic acid is a BA and its levels are reportedly increased in chronic kidney disease patients (Wu et al., 2020). In our research, we found that the content of glyco-Lambda-muricholic acid increased in T2DM patients. The cAMP signaling pathway, which regulates glucose homeostasis, has recently been recognized to regulate insulin secretion, glycogen synthesis, gluconeogenesis, etc. (Yang and Yang, 2016). Compared with the Mod group, STG may target the cAMP signaling pathway for the treatment of T2DM. Tryptophan metabolism is strongly related to the onset and progression of T2DM (Sudar-Milovanovic et al., 2022). The serum tryptophan concentration was lower in the T2DM group than in the control group, which was part of the effect of the STG intervention.

BAs are essential physiological agents involved in nutrient absorption, partitioning, metabolism and excretion (Fogelson et al., 2023; Hou et al., 2023). Primary BAs are among the metabolic products of cholesterol breakdown in the liver. Secondary BAs are released from the gallbladder into the intestines, where they are catalyzed and converted by a series of enzymes (Ridlon et al., 2006). Moreover, BAs are also important components maintaining the function of the intestinal mucosa and have regulatory effects on the intestinal mucosal barrier and metabolite function (Zhang et al., 2021; Shi et al., 2023). Previous studies have shown higher total BAs concentrations in patients with T2DM than in healthy individuals, and BA metabolism is closely related to glucose metabolism (Haeusler et al., 2013; Sun et al., 2020). The main BAs associated with BA biosynthesis are beta-muricholic acid, alpha-muricholic acid, glycochenodeoxycholic acid, ursocholic acid, glycohyodeoxycholic acid and dioxolithocholic acid. Wang et al. (2022) reported that differentially abundant metabolites in the faeces of T2DM model rats could also be enriched for BA biosynthesis, and beta-muricholic acid was one of the metabolites. In our research, beta-muricholic acid was considered a potential biomarker for diabetes, and it is closely linked to FBG levels and the HOMA-IR. Elevated glycochenodeoxycholic acid levels may be a risk factor for hepatic steatosis and hepatic fibrosis in T2DM patients (Forlano et al., 2024). Cholic acid methyl ester has potential beneficial effects on T2DM (Mikov et al., 2006). Since GK rats mainly exhibit different degrees of steatosis, these findings are consistent with the results of the present study. Moreover, STG can decrease the levels of the BAs described above and remodel the metabolism of BAs in the ileal contents to some extent.

Current research shown that an imbalance of intestinal microorganisms and metabolites is a characteristic of T2DM. STG can play a role in reducing blood glucose levels, and underlying mechanism could be related to alleviating this imbalance. A new application of a traditional Chinese medicine for regulating blood glucose levels via effects on the gut microbiota and metabolites has been developed.

## Data availability statement

The data presented in the study are deposited in the NCBI repository, accession number: PRJNA1144618.

## Ethics statement

The animal studies were approved by the Anhui Chinese Medicine University. The studies were conducted in accordance with the local legislation and institutional requirements. Written informed consent was obtained from the owners for the participation of their animals in this study.

## Author contributions

J-DZ: Writing – original draft. Z-HF: Writing – review & editing.

## Footnotes

^1^ https://www.metaboanalyst.ca/

^2^ https://meta.bgi.com/microbe/login

^3^ https://www.omicshare.com/
